# Theory-based training to promote breast cancer screening among women with breast cancer worries: randomized controlled trial

**DOI:** 10.1590/1516-3180.2019.033430092019

**Published:** 2020-06-01

**Authors:** Sermin Timur Taşhan, Yeşim Aksoy Derya, Tuba Uçar, Gülçin Nacar, Behice Erci

**Affiliations:** I PhD. Professor, Department of Birth, Women’s Health and Illness, Faculty of Nursing, Inönü Üniversitesi, Malatya, Turkey.; II PhD. Associate Professor, Department of Midwifery, Faculty of Health Sciences, Inönü Üniversitesi, Malatya, Turkey.; III PhD. Associate Professor, Department of Midwifery, Faculty of Health Sciences, Inönü Üniversitesi, Malatya, Turkey.; IV PhD. Research Assistant, Department of Birth, Women’s Health and Illness, Faculty of Nursing, Inönü Üniversitesi, Malatya, Turkey.; V PhD. Professor, Department of Public Health, Faculty of Nursing, Inönü Üniversitesi, Malatya, Turkey.

**Keywords:** Breast neoplasms, Mammography, Breast self-examination, Early diagnosis, Health-promoting behaviors, Health belief model, Breast cancer screening training

## Abstract

**BACKGROUND:**

Breast cancer worries are important determinants in relation to behavior favoring breast cancer screening.

**OBJECTIVE:**

To determine the effect of theory-based training to promote breast cancer screening among women with high and low levels of breast cancer worries.

**DESIGN AND SETTING:**

Randomized controlled trial, conducted in two family health centers.

**METHODS:**

In total, 285 women were recruited. Women with low levels of breast cancer worries were included in the first intervention group (112 women) and the first control group (112 women), while women with high levels of breast cancer worries were included in the second intervention group (37 women) and the second control group (43 women). Theory-based training to promote breast cancer screening was given to intervention groups. The women’s willingness to undergo breast cancer screening and breast cancer worry scores were evaluated at 1, 3 and 6 months.

**RESULTS:**

The women in the low cancer-worry intervention group performed breast self-examination more in months 1 and 6 following the training, and the women in the high cancer-worry control group performed breast self-examination more in month 3 (P < 0.05). No difference between the women who had low or high levels of breast cancer worries were observed in relation to breast self-examination, clinical breast examination or mammography (P > 0.05).

**CONCLUSION:**

The level of worry did not affect the success of theory-based training, and the training was partially effective with regard to willingness to undergo breast cancer screening.

## INTRODUCTION

Breast cancer is the most frequent type of cancer and the most common cause of cancer death among gynecological cancers. One in every four women with cancer in the world has breast cancer. The International Cancer Agency reported that there were around 2,088,849 new cases and 626,679 deaths due to breast cancer worldwide in 2018.^1^ The incidence of breast cancer is higher in developed countries than in developing countries, but the numbers of deaths due to breast cancer are lower in developed countries than in developing countries.^2^^,^^3^

It is known that breast self-examination, clinical breast examination and mammography play an important role in making an early diagnosis of breast cancer. The uptake rate for mammography performed on a regular basis is low because this is an expensive method, considering that not all individuals have health insurance and public funding is inadequate, especially in developing countries. Hence, breast self-examination (which has no cost) and clinical breast examination (which only has low cost) remain important diagnostic methods. Moreover, during clinical breast examination, healthcare professionals have the opportunity to advise on breast cancer, risk factors, prevention methods and screening methods.^4^^,^^5^^,^^6^

Awareness of the barriers relating to willingness to undergo breast cancer screening is important. Azami-Aghdash et al. found that the biggest barriers impeding willingness to participate in breast cancer screening programs were lack of information, problems regarding transportation to the clinic and fear, in decreasing order.^7^ In a study conducted by Tuzcu and Bahar in Turkey, lack of information was found to be the primary factor preventing willingness to undergo breast cancer screening.^8^ Several studies in the literature have investigated the effect of education for overcoming the barrier of lack of information on breast cancer screening.^9^^,^^10^^,^^11^

The concept of cancer can cause fear or worry. This fear is the third largest barrier against undergoing breast cancer screening and can direct women’s behavior in this regard. Fear or worry about getting cancer can sometimes make women more willing to look for early diagnosis, but sometimes it can be a deterrent.^11^ There are results in the literature indicating that negative emotions such as fear and worry about health problems can effectively lead people to avoid seeking early diagnosis relating to cancer.^13^^,^^14^^,^^15^^,^^16^ Examination of women’s worries regarding breast cancer and their behavioral decisions during follow-up should be the focal point of personal education relating to cancer.^12^^,^^17^^,^^18^

So far, the effects of fear and worries about cancer on women’s learning process and behavior regarding breast cancer screening have only been addressed in a limited manner. It is expected that the present study will make a significant contribution towards better understanding of women’s attitudes and tendencies towards breast cancer screening.

## OBJECTIVE

This study was conducted to determine the effect of theory-based training to promote breast cancer screening among women with breast cancer worries. In addition, behavior regarding breast cancer screening was compared between women with high and low levels of worry about breast cancer.

## METHODS

### Study design, setting, participants and ethics

A randomized controlled trial was conducted at two family health centers providing primary health care services at locations in eastern Turkey. The population for this study consisted of 3,900 women aged 20-65 years who were registered at these family health centers.

A power analysis was conducted to determine the sample size, through calculations using the publicly available statistical software OpenEpi, version 3 (http://www.openepi.com). This analysis was done using a significance level of 5%, an effect size of 22% and an ability to represent the population of 80% (power). It was shown that the sample size needed to be at least 105 women in each group (i.e. 105 in the intervention group and 105 in the control group).

Regarding randomization and allocation concealment, women for the control groups were selected from Başharık family health center and women for the intervention groups were chosen from Sıtmapınarı family health center. These women were recruited from both family health centers using simple random sampling. A random number table was used at each family health center, which enabled recruitment of 1,530 women.

The Breast Cancer Worry Scale (BCWS) was administered to 420 women who met the inclusion criteria. Women who were found to have low levels of worries about breast cancer were included in the first intervention group and the first control group, while women with high levels of worries about breast cancer were included in the second intervention group and the second control group. Totals of 305 women (intervention 182; control 123) with low levels of worries about breast cancer and 115 women (intervention 55; control 60) with high levels of worries about breast cancer were identified according to their BCWS scores.

**Figure 1.**
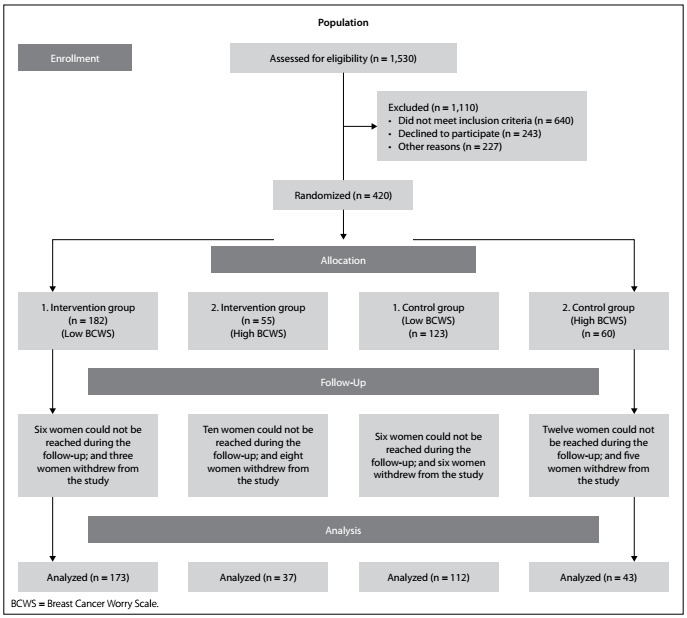
CONSORT (Consolidated Standards of Reporting Trials) flowchart for the study.

After allocation, no blinding for group assignment was possible for either the participants or the researchers. This was because follow-up interviews were conducted between the women and researchers. The study protocol was completed by 173 women in the low breast cancer-worry intervention group and 112 women in the low breast cancer-worry control group (a total of 285); and by 37 women in the high breast cancer-worry intervention group and 43 women in the high breast cancer-worry control group (a total of 80). These smaller numbers were because some women wanted to withdraw from the study (n = 22) and some changed their address (n = 33) during the data collection phase (Figure 1).

The inclusion criteria were as follows. The participants included did not have any diagnosis of breast cancer, had not been performing breast self-examination regularly (every month), had not previously had a mammogram, had not previously had a clinical breast examination, were not pregnant or breastfeeding and were literate.

According to the breast cancer screening program of Turkey, women aged 20 years and over should perform breast self-examination every month; women aged 20 years and over should undergo clinical breast examination once every two years; women aged 40 years and over should undergo clinical breast examination once a year; and women aged 40-69 years should undergo mammography every year.^21^ Therefore, women who had been doing breast self-examination once a month were accepted as performing breast self-examination. Among women aged 40 years and over, at least one clinical breast examination within the first six months after training and having a mammogram were accepted as having undergone clinical breast examination and mammography. The Sıtmapınarı and Başharık family health centers serve the largest populations around the provincial border of Malatya (Sıtmapınarı family health center serves 2,500 women and Başharık family health center serves 1,400 women), and the populations that they serve present sociodemographic homogeneity.

### Ethics

This study was endorsed by the Internal Review Board (Ethics Committee) of Inönü Üniversitesi on April 16, 2014, under the approval number 2014/44. This study was registered in the Clinical Trial Registry (NCT04225741).

### Measurements

Data were collected using a personal information form, a breast cancer screening behavior questionnaire (BCSBQ) and the BCWS, between January 2015 and August 2017.

*Personal information form*: This form, prepared by the researchers, consisted of questions regarding the sociodemographic characteristics of the women.

*Breast Cancer Screening Behavior Questionnaire*: This questionnaire, prepared by the researchers, comprised questions concerning breast self-examination, clinical breast examination and mammography practices.^19^ No validated tool for assessment of breast cancer screening behavior was available in Turkey. The BCSBQ was prepared in line with the national standards that need to be followed during breast cancer screening program studies conducted by the Turkish Ministry of Health.^19^

*Breast Cancer Worry Scale:* Lerman et al.^20^ developed this three-item scale to measure breast cancer worry levels and their effect on daily activities and mood. Lerman subsequently modified the scale, such that it was extended from breast cancer to general cancer and its number of questions was increased to six.^20^ Lerman’s six-item cancer worry scale was then modified by Timur Taşhan et al. to measure breast cancer worries alone, and a Turkish validity and reliability study on the BCWS was conducted. This Turkish-language validated version of the BCWS uses a five-item Likert-type scale, and for each question on this scale, respondents need to choose one of the following options: never = 0, rarely = 1, sometimes = 2, often = 3, or always = 4. Thus, overall, the lowest score that can be obtained is 0, and the highest is 24. A total score of less than 12 denotes a low level of worry regarding cancer, and a total score ≥ 12 indicates a high level of worry.^21^ Cronbach’s alpha reliability coefficient for the Turkish-language validated version of the BCWS was 0.78.

### Procedure

Written permission to conduct the study was obtained from the Public Health Institution of Turkey and from the Sıtmapınarı and Başharık family health centers. In addition, approval was obtained from Inonu University Health Sciences Scientific Research and Publication Ethics Committee (April 16, 2014, under number 44). Before beginning the study, verbal consent was obtained from all the women who participated. The intervention and control group data were collected simultaneously. After making appointments with the women by phone, the data were collected by the researchers in four stages in the women’s own homes, using face-to-face interviews.

The BCWS and the personal information form were administered to the women who had been selected to form the two control groups, during the first interview, in order to determine breast cancer-worry levels*.* Following this first interview, follow-up interviews were conducted one, three and six months later, and the BCSBQ was administered at each follow-up appointment.

 Following administration of the BCWS and the personal information form to the women who had been selected to form the two intervention groups (a low breast cancer-worry group and a high breast cancer-worry group), during the first interview, the researchers gave the breast cancer screening training to both intervention groups under equal conditions in the training room of Sıtmapınarı family health center, in the form of group training (8-12 women). Following this training, the women in the intervention groups received consultations at follow-ups, via home visits in months 1, 3, and 6. At these times, the researchers administered the BCSBQ.

The primary outcome measurement of this study was the efficacy of the theory-based training on breast cancer screening behavior. The secondary outcome measurements were changes to breast cancer screening behavior.

### The intervention

The single-session training lasted for approximately 40-45 minutes and was conducted in the training room of Sıtmapınarı family health center, as a suitable environment. The health belief model predicts the determinants of preventive health behaviors and explains inadequate participation in disease prevention and screening programs.^22^^,^^23^ Furthermore, this model not only explains behavior regarding screening, but also evaluates the cognitive factors that facilitate health-promoting behaviors.^22^^,^^23^^,^^24^

Many previous studies have simultaneously examined the health belief model and behavior favoring breast cancer screening.^22^^,^^25^^,^^26^^,^^27^^,^^28^ Therefore, this model was used in the training provided in the present study, with the aim of achieving better comprehension among the participants regarding the importance of screening for breast cancer. Through this training, participants would acquire the ability to correctly perform breast self-examination and would understand the necessity for mammography and clinical breast examination, in accordance with the health belief model. The following notions were addressed:

- Perceived susceptibility: In order to increase the women’s perception of susceptibility to breast cancer, explanations of the disease and its epidemiology, the structure of the breast and breast cancer risk factors were provided.
- Perceived severity: In order to increase the women’s perception of the severity of breast cancer, the characteristics of breast lumps, as diagnosed in early and late breast cancer, and the differences in the treatment regimens were explained.
- Perceived benefit: In order to improve the women’s perception of breast cancer screening, the treatment benefit of early diagnosis of breast cancer, the role of alternative treatment methods, such as lumpectomy instead of radical mastectomy, and the effect of regularly performed examinations on the breast cancer mortality rate were explained.
- Perceived trust: How to correctly conduct breast self-examination, what clinical breast examination consists of, why mammography is performed and how long it takes to perform mammography were explained.
- Perceived barrier: In order to reduce the women’s perceived barriers against undergoing breast cancer screening, the factors inhibiting women from conducting breast self-examination and from undergoing clinical breast examination and mammography were explained in detail.

None of the interventions described above were applied to the control group.

### Statistical analysis

The data were evaluated using the Statistical Package for the Social Sciences software, version 16.0. In the data assessment, percentages, means, independent-sample t tests, chi-square tests, Fisher’s exact tests and repeated-measurement analysis of variance (ANOVA) tests were used. To compare the groups regarding categorical variables, the chi-square test and Fisher’s exact test were used. An independent t test was used to make comparisons between the intervention and control groups. To test for a significant difference in means over time, repeated-measurement ANOVA was used. The statistical significance level was taken to be P < 0.05.

## RESULTS

The age, employment status, marital status, educational level and economic level of the intervention and control groups were similar. No statistically significant difference was found between the intervention and control groups in terms of sociodemographic characteristics (Table 1).

**Table 1.** Sociodemographic characteristics of the women in the intervention and control groups

Characteristics	Experimental group (n = 210)		Control group (n = 155)		χ^2^	P
	n	%	n	%		
Age (years)						
< 40	90	42.9	53	34.2	2.809	0.094
≥ 40	120	57.1	102	65.8		
Employment status						
Unemployed	159	75.5	114	73.5	0.222	0.638
Employed	51	24.5	41	26.5		
Marital status						
Married	174	82.9	137	88.4	2.163	0.141
Single	36	17.1	18	11.6		
Educational level						
Literate	32	15.3	21	13.5	7.030	0.071
Primary school	75	35.7	76	49.0		
Secondary/high school	58	27.6	30	19.4		
University	45	21.4	28	18.1		
Economic level						
Low	115	54.8	79	51.0	0.516	0.473
Medium	95	45.2	76	49.0		

The mean BCWS scores of the women in the intervention group with low levels of cancer worries increased gradually from the pre-intervention test to the tests in months 1, 3 and 6, and the differences in the scores were statistically significant (P = 0.001). No difference in the mean BCWS scores between the pre-test and the tests in months 1, 3 and 6 was observed among the women in the control group with low levels of cancer worries (P = 0.096). There was no difference in the mean BCWS scores between the pre-test and the tests in months 1, 3 and 6 among the women in the intervention group with high levels of cancer worries (P = 0.263). The mean BCWS scores of the women in the control group with high levels of cancer worries decreased gradually from the pre-test to the tests in months 1, 3 and 6, and the differences in the scores were statistically significant (P = 0.001) (Table 2).

**Table 2.** Comparison of breast cancer-worry levels among the women in the intervention and control groups

LBCWS	Low breast cancer-worry intervention group (n = 173)	Low breast cancer-worry control group (n = 112)	t	P
	Mean ± SD	Mean ± SD		
Pre-test	3.70 ± 3.36	4.58 ± 3.61	2.100	0.037
Month 1	4.11 ± 3.56	4.75 ± 3.59	1.620	0.106
Month 3	4.25 ± 3.60	4.03 ± 3.42	0.284	0.777
Month 6	4.74 ± 3.41	4.40 ± 3.15	0.657	0.512
F	9.680	2.167		
P	0.001	0.096		
HBCWS	High breast cancer-worry intervention group (n = 37)	High breast cancer-worry control group (n = 43)	t	P
	Mean ± SD	Mean ± SD		
Pre-test	14.72 ± 3.51	14.93 ± 3.01	-0.269	0.788
Month 1	13.51 ± 4.22	12.30 ± 4.25	1.265	0.210
Month 3	13.05 ± 3.12	11.41 ± 5.16	1.622	0.109
Month 6	12.50 ± 3.91	10.6 ± 5.47	1.827	0.071
F	2.668	6.318		
P	0.263	0.001		

LBCWS = low breast cancer-worry scale; SD = standard deviation; HBCWS = high breast cancer-worry scale.

With regard to the women with low levels of breast cancer worries, it was found that 41.6% of the women in the intervention group and 20.5% of the women in the corresponding control group performed breast self-examination in the first month after receiving the theory-based training. This difference in use of breast self-examination was statistically significant (P = 0.001). In addition, 56.1% of the women in the intervention group and 42% of the women in the control group performed breast self-examination in month 6, which was a statistically significant difference (P = 0.021). No differences in the rates of performing breast self-examination in the third month or undergoing clinical breast examination and mammography within the first six months after training were found between the women in the intervention and control groups (Table 3).

**Table 3.** Comparison of breast cancer screening behaviors among the women in the intervention and control groups who presented low levels of cancer worry

Breast cancer screening behaviors	Intervention group (n = 173)		Control group (n = 112)		χ^2^	P
	n	%	n	%		
Month 1 BSE						
Yes	72	41.6	23	20.5	13.598	0.001
No	101	58.4	89	79.5		
Month 3 BSE						
Yes	91	52.6	50	44.6	1.723	0.189
No	82	47.4	62	55.4		
Month 6 BSE						
Yes	97	56.1	47	42.0	5.411	0.021
No	76	43.9	65	58.0		
CBE						
Yes	24	13.9	17	15.2	0.094	0.759
No	149	86.1	95	84.8		
Mammography^a^ (n = 170)						
Yes	8	8.2	6	8.2		0.995^b^
No	89	91.8	67	91.8		

^a^Women aged 40 years and over were evaluated; ^b^Fisher’s exact test was used. BSE = breast self-examination; CBE = clinical breast examination.

With regard to the women with high levels of breast cancer worries, it was observed that 45.9% of the women in the intervention group and 79.1% of the women in the control group performed breast self-examination in month 3 after training. This difference in use of breast self-examination was statistically significant (P = 0.020). No differences in the rates of performing breast self-examination in months 1 and 6 or having clinical breast examination and mammography within the first six months were found between the women in the intervention and control groups (Table 4).

**Table 4.** Comparison of breast cancer screening behaviors of the women in the intervention and control groups who presented high levels of breast cancer worry

Breast cancer screening behaviors	Intervention group (n = 37)		Control group (n = 43)		P
	n	%	n	%	
Month 1 BSE					
Yes	16	43.2	18	41.9	0.901
No	21	56.8	25	58.1	
Month 3 BSE					
Yes	17	45.9	34	79.1	0.020
No	20	54.1	9	20.9	
Month 6 BSE					
Yes	18	48.6	30	69.8	0.054
No	19	51.4	13	30.2	
CBE					
Yes	9	24.3	10	23.3	0.911
No	28	75.7	33	76.7	
Mammography^a^ (n = 52)					
Yes	-	-	3	-	
No	23	100.0	26	100.0	

^a^Women aged 40 years and older were evaluated; P: Fisher’s exact test was used.

BSE = breast self-examination; CBE = clinical breast examination.

## DISCUSSION

Encouraging women to have cancer screening tests on a regular basis is an important requirement in the fight against breast cancer. However, a variety of psychosocial factors affect behaviors such as willingness to undergo cancer screening tests.^3^ Cancer-related thoughts can result in various negative reactions, such as anxiety, fear and grief.^11^^,^^29^ Fear or worry about getting cancer is the most prevalent of these psychosocial factors.^11^ In this context, studies on the types of differences that psychosocial factors show with regard to willingness to seek early diagnosis, depending on cultural structures, are required.^30^ The present study was conducted to determine the effect of theory-based training given to women, on the basis of their breast cancer-worry level, on their behavior towards breast cancer screening.

The results from the follow-ups conducted in months 1, 3 and 6 showed that the breast cancer worries of women in the low breast cancer-worry intervention group gradually and significantly increased. In contrast, the breast cancer worries of the women in the high breast cancer-worry control group gradually and significantly decreased (P < 0.05).

Janz et al. reported that worry about cancer recurrence led individuals to ask more questions at consultations with their doctors.^23^ It has also been stated that there is a high possibility that individuals will follow the recommendations of people in whom they place a high degree of trust, such as doctors and clergymen.^30^^,^^31^ Çaman et al. observed that the advice of physicians was effective in encouraging women to visit cancer screening centers. These authors also revealed that the actions of healthcare professionals were an important factor with regard to affecting women’s levels of worry.^32^

In the present study, breast cancer risk factors, the characteristics of the lump and the differences in the treatment regimens used, depending on whether breast cancer is diagnosed at an early or late stage, were explained under the headings of perceived susceptibility and perceived severity, in accordance with the basic components of the health belief model.^18^^,^^25^ This information was thought to result in an increase in the level of worry among the women in the low cancer-worry intervention group, but in a decrease in the level of worry among the women in the high breast cancer-worry control group. The increase in the level of worry in this intervention group was attributed to forgetting the information over time.

A difference favoring the low cancer-worry intervention group in months 1 and 6, in terms of breast self-examination, was identified. However, this difference favored the high cancer-worry control group with regard to breast self-examination in month 3. Kim et al.^33^ found that women with high levels of cancer worries had unrealistic pessimism. Negative beliefs surrounding cancer treatment or survival may mean that they do not want to know about the cancer in advance, and this can negatively affect their behavior in relation to obtaining early diagnosis of cancer.^11^ Gasalberti showed that breast cancer worries were a barrier to carrying out breast self-examination,^34^ while Arts-de Jong et al.^35^ found a correlation between demoralization and cancer worries. The results from the present study are concordant with the results from these previous studies.

Although some previous studies on the effects of training on women’s willingness to undergo breast cancer screening indicated that this training did not have any effect in relation to clinical breast examination^9^ or mammography,^8^^,^^9^ other studies have shown that training has a significant effect on willingness to perform breast self-examination^9^^,^^10^ and to undergo clinical breast examination and mammography.^10^ In a study on cervical cancer conducted by Ngua et al.,^36^ it was found that the training given had no effect in month 6. In the present study, it was shown that the training provided had a short-term effect on the women’s behavior, and that this effect was mainly in relation to breast self-examination. It was observed that the training given and the cancer-worry level had no effect on willingness to undergo clinical breast examination and mammography, which are the diagnostic methods that provide the most valuable results. This finding partially supports the hypothesis that “theory-based training does not affect women’s acquisition of behavior favoring breast cancer screening”. The results from the present study are similar to those of previous studies in this regard.

Numerous studies have found that cancer risk perception and worries about getting cancer are two important variables that have mutual interaction.^3^^,^^37^^,^^38^^,^^39^ In this context, the effects of both breast cancer worries and breast cancer risk perception on willingness to undergo breast cancer screening have been investigated. While some studies showed that behavior favoring breast cancer screening increased as the worry or risk perception increased,^38^^,^^40^^,^^41^^,^^42^^,^^43^^,^^44^ one other study found that there was no difference.^45^ Baysal and Gozum^46^ found that a higher uptake rate for mammography was associated with low levels of breast cancer risk. There was no difference in the rates of breast self-examination, clinical breast examination and mammography practices between intervention groups with low and high levels of cancer worry. This finding supports the hypothesis that “the level of breast cancer worry among women does not affect the acquisition of behavior favoring breast cancer screening.”

Amuta et al.^47^ stated that this worry had a short-term effect on health-related behavior and that such behavior also changed when there was no emotion in making decisions regarding health. In addition, these authors found that cancer worries did not affect the frequency of attending cancer screenings. Çaman et al.^32^ conducted a study in the Early Diagnosis, Screening and Education Center for Cancer of Turkey and found that there was no statistically significant correlation between cancer risk perception and breast self-examination frequency. In addition, no significant correlation was found between the thought of participating in breast cancer screening programs in the future and cancer risk perception. Seven et al.^39^ found that there were no correlations between women’s perception of risk with regard to getting breast cancer and their level of knowledge about breast cancer, doing breast self-examination and undergoing mammography. The results from the present study are concordant with the results reported by Amuta et al.,^32^ Çaman et al.^39^ and Seven et al.^47^

The first limitation of this study was the low number of women included who had high levels of breast cancer worry. The second was that the education given to the women in the experimental group was presented as group-based education. And lastly, the levels of pre- and post-training knowledge and the actual risks of breast cancer among these women were not assessed.

## CONCLUSIONS

It was found in the present study that theory-based training had a partial effect on willingness to perform breast self-examination and no effect on willingness to undergo clinical breast examination and mammography. In addition, it was observed that the worry level of the women had no effect on the success of theory-based training to promote breast cancer screening. It is thought that informing these women about the risk factors for acquiring breast cancer screening behaviors caused them to worry, but that their worry did not affect their behavior. Rather, it gave them more positive messages and, therefore, investigation of the effect of this approach on breast cancer screening behavior is required.
